# Photosynthetic and Canopy Characteristics of Different Varieties at the Early Elongation Stage and Their Relationships with the Cane Yield in Sugarcane

**DOI:** 10.1155/2014/707095

**Published:** 2014-06-22

**Authors:** Jun Luo, Yong-Bao Pan, Liping Xu, Yuye Zhang, Hua Zhang, Rukai Chen, Youxiong Que

**Affiliations:** ^1^Key Laboratory of Sugarcane Biology and Genetic Breeding, Ministry of Agriculture, Fujian Agriculture and Forestry University, Fuzhou 350002, China; ^2^USDA-ARS, Sugarcane Research Laboratory, Houma, LA 70360, USA

## Abstract

During sugarcane growth, the Early Elongation stage is critical to cane yield formation. In this study, parameters of 17 sugarcane varieties were determined at the Early Elongation stage using CI-301 photosynthesis measuring system and CI-100 digital plant canopy imager. The data analysis showed highly significant differences in leaf area index (LAI), mean foliage inclination angle (MFIA), transmission coefficient for diffused light penetration (TD), transmission coefficient for solar beam radiation penetration (TR), leaf distribution (LD), net photosynthetic rate (PN), transpiration rate (*E*), and stomatal conductance (GS) among sugarcane varieties. Based on the photosynthetic or canopy parameters, the 17 sugarcane varieties were classified into four categories. Through the factor analysis, nine parameters were represented by three principal factors, of which the cumulative rate of variance contributions reached 85.77%. A regression for sugarcane yield, with relative error of yield fitting less than 0.05, was successfully established: sugarcane yield = −27.19 − 1.69 × PN + 0.17 ×  *E* + 90.43 × LAI − 408.81 × LD + 0.0015 × NSH + 101.38 ×  *D* (*R* ^2^ = 0.928**). This study helps provide a theoretical basis and technical guidance for the screening of new sugarcane varieties with high net photosynthetic rate and ideal canopy structure.

## 1. Introduction

Photosynthesis is the basis of yield formation. It is generally recognized that about 90%–95% of crop yield is formed from the assimilated carbons. Therefore, how to improve crops' photosynthetic capacity is one of the most important targets in the studies of genetics and breeding [1–4]. The canopy structure of a crop should be optimized so that an increased proportion of light can reach the leaves at the base of the plants [5, 6]. Studies on photosynthetic characteristics of crops such as* Zea mays* [7–9],* Oryza sativa* [10–12], and* Glycine max* [13, 14] have been extensively performed. Also, inheritance of photosynthetic characteristics [15–17], diurnal variation of net photosynthetic rate [18, 19], and seasonal variation [20, 21] have been reported in the separated sugarcane seedling populations. Indices such as shape, size, quantity, and spatial distribution of leaves are directly related to the light environment and light utilization rate of the population. They are the important factors affecting the light distribution in crop population and photosynthesis [22].

Optimizing a reasonable population structure, improving the light distribution in the population, and increasing the rate of light utilization are all effective ways in obtaining high crop yields. Leaf area index (LAI) determines canopy light interception, which affects the photosynthetic rate of a crop population. When LAI is at the optimal level, canopy light interception reaches the highest level for improved photosynthetic capacity and potential yield increase [23]. The relations between sugarcane leaf morphology and cane yield and between sucrose content and transpiration rate of leaves have been studied [24–27]. However, studies on the canopy characteristics of sugarcane were rarely reported [28, 29], although there are many yield-related traits and indices controlling photosynthesis and canopy structure of sugarcane. The interactions between these indices are complicated due to the influences of genetic characteristics, environmental factors, and sample selections. The factor analysis method allows the categorization of a large number of correlated agronomic traits into several groups based on principal factors. It has been widely used in studies of crop germplasm resources. In sugarcane, a correlation analysis of seasonal variation of the canopy structure parameters to yield-related traits was conducted [21]; however, there has been no report on the application of factor analysis to the classification of photosynthetic and canopy parameters and their effects on crop yield in sugarcane [30, 31].

During sugarcane growth, Early Elongation stage is a critical period for cane yield formation. During this period, an appropriate number of stalks per unit area are controlled through timely cultivation to achieve high yield [25]. In this study, we aimed to investigate the effects of photosynthetic and canopy characteristics of 17 sugarcane varieties at the Early Elongation stage on cane yield. The results of this study would provide the scientific basis for breeding of sugarcane varieties with high photosynthetic efficiency and for improving cultivation technique. Hierarchical cluster analysis was performed by the numerical classification method on the photosynthetic and canopy parameters of the involved sugarcane varieties. The relationship and the stepwise regression analysis between canopy and photosynthetic characteristics and cane yield were also conducted, in order to screen new sugarcane varieties with high net photosynthetic rate (PN) and ideal canopy structure.

## 2. Materials and Methods

### 2.1. Sugarcane Varieties

Seventeen sugarcane varieties were tested, namely, FN94-0403, FN94-0744, FN95-1726, FN96-0907, FN98-10100, ROC10, GT94-116, GT95-118, GT96-211, GT96-44, GT97-18, MT70-611, YT92-1287, YT96-107, YT96-794, YT96-835, and YT96-86.

### 2.2. Field Experiment Design and Cane Yield Traits

Field experiment was conducted on the Experimental Farm of the Key Laboratory of Sugarcane Biology and Genetic Breeding, Ministry of Agriculture/Fujian Agriculture and Forestry University located at Jianxin, Cangshan District, Fuzhou, Fujian (longitude: 119.23E, latitude: 26.08N). A randomized block design with three replications was adopted. Plot area was 33 m^2^, with three rows at 1.1 m row spacing. Planting density was 45,000 two-bud setts ha^−1^. The preceding crop was sugarcane, and the soil type was sandy loam soil. The nutrients of the arable layer before sowing included organic matter of 10.5 g/kg, total nitrogen of 0.91 g/kg, alkaline hydrolysis nitrogen of 90.01 mg/kg, available phosphorus of 110.4 mg/kg, and rapidly available potassium of 369.5 mg/kg. The rows were covered by plastic films after planting. Planting was performed in a scheme of double buds and double rows. Herbicides were applied before planting and plastic film mulching. Weed control was repeatedly performed as needed. The basal fertilizer Calcium Super Phosphate at 750 kg ha^−1^ was applied during planting. During growing season, 975 kg ha^−1^ and 600 kg ha^−1^ KCl were applied for two times as topdressing. Field management level was only slightly more intensive than that of the local standard operations on cultivation, fertilization, irrigation, and pest control. Each application was completed covering all the experimental plots on the same day.

In Fuzhou, sugarcane elongation period lasts from late June to early November, while Early Elongation stage commences from late June and ends in early July. Measurements of photosynthetic characteristics and canopy parameters were conducted in early July when sugarcane seedlings reached an average height of 80.9 cm. Data on yield-related traits, including plant height (*H*), stalk diameter (*D*), single stalk weight (SSW), and the number of effective stalks per hectare (NSH), were collected before harvesting. All the stalks in the middle row of each plot were cut and weighed. The sampled sugarcane areas were measured. The number of millable stalks within the sampling area was also counted. Single stalk weight and cane yield were calculated by the following formulae [25]:

 Single stalk weight = (plant height × stalk diameter^2^ × 0.785)/1000; Cane yield = single stalk weight × the number of effective stalks per hectare.

### 2.3. Determination of Photosynthetic Parameters

Photosynthetic parameter data were collected between 8:30–11:30 AM on a sunny morning in early July, using a CI-301 photosynthesis system (CID Co., Ltd, Vancouver, WA, USA) under natural light conditions. The data included net photosynthetic rate (PN, *μ*molm^−2^ s^−1^), transpiration rate (*E*, mmolm^−2^ s^−1^), and stomatal conductivity (GS, mmolm^−2^ s^−1^). The PN equals the rate of photosynthetic CO_2_ fixation minus the rate of CO_2_ loss during respiration. The *E* is the amount of evaporation per unit time from a leaf surface. The GS is the rate of CO_2_ entering stomata [17, 22]. The measurements were taken three times on each variety following the protocol described earlier with minor modifications [17, 22, 31]. The first youngest fully expanded (+1) leaves at the top canopy were measured reciprocally at the middle to upper section excluding the midrib. The direction of leaf chamber was adjusted towards sunlight to ensure that measurements were done under a uniform light intensity. A total of 18 plants were measured for each variety under the following natural conditions: light intensity (1,781.17 ± 103.21 *μ*molm^−2^ s^−1^), ambient temperature (T_*a*_) (28.07 ± 2.44°C), leaf temperature (30.14 ± 2.26°C), ambient relative humidity (RH) (47.71 ± 4.88%), and ambient CO_2_ concentration (C_*a*_) (331.02 ± 25.35 *μ*L·L^−1^).

Several parameters were recorded simultaneously, including effective photosynthetic radiation (PAR), relative humidity (RH), ambient temperature (T_*a*_), ambient CO_2_ concentration (C_*a*_), stomatal conductivity (GS), evaporation rate (*E*), and between cell CO_2_ concentrations (C_*i*_). Because all determinations were done under the natural sunlight, only those measurements were kept when natural light intensity is at 1,600 *μ*mol/M^2^·Sec, while measurements taken under 1,600 *μ*mol/M^2^·Sec light intensity were discarded. CO_2_ concentrations were collected from the upper level of canopy after passing through the dual-bottle apparatus and were slightly lower than those collected above the ground level. This is due to the fact that sugarcane is a highly efficient C_4_ crop. Only mean values and standard deviations were shown in the text.

### 2.4. Determination of Canopy Parameters

A fisheye lens of the CI-100 digital plant canopy imager (CID Co., Ltd, Vancouver, WA, USA) was attached to an observation rod and placed in the center of the rows in a cloudless twilight afternoon with a little sunshine in early July. The rod was adjusted to the horizontal direction for taking photos when there was no shadow or any other external influences. Fifteen testing points were selected for each variety and five images were taken from each plot. LD was represented by distribution frequency of the leaf within each azimuth. The canopy indicators such as leaf area index (LAI), mean foliage inclination angle (MFIA), transmission coefficient for diffuse light penetration (TD), transmission coefficient for solar beam radiation penetration (TR), and leaf distribution (LD) were calculated by Plant Canopy Analysis software provided by CID Company (CID Co., Ltd, Vancouver, WA, USA) [21].

### 2.5. Statistical Methods

Variance analysis, cluster analysis, factor analysis, and regression analysis were performed using DPS software (Zhejiang University, Hangzhou, China) [30]. After standardization of original data, the distance coefficient was determined as the chi-square distance and cluster analysis by Ward's method [30]. Varimax rotation was used to change the coordinates used in principal component analysis and factor analysis [21, 30]. A stepwise regression analysis was also conducted for the photosynthetic and canopy parameters and their correlation with cane yield [21, 30].

## 3. Results

### 3.1. Estimated Cane Yield Trait Data

Estimated cane yield trait data are shown in Table 1.

### 3.2. Differences in Photosynthetic Parameters among 17 Sugarcane Varieties

There were extremely significant differences in the mean PN, *E*, and GS among the 17 sugarcane varieties (Table 2). Based on PN, the 17 sugarcane varieties were classified into four categories. Category I (high PN) included five varieties, namely, FN98-10100, GT94-116, GT96-211, YT96-794, and YT96-86, with a mean PN value of 37.09 *μ*molm^−2^ s^−1^. Category II included four varieties (FN94-0403, FN95-1726, FN96-0907, and YT96-107) with a mean PN value of 35.68 *μ*molm^−2^ s^−1^. Category III included six varieties (GT95-118, GT97-18, MT70-611, ROC10, YT92-1287, and YT96-835) with a mean PN value of 34.24 *μ*molm^−2^ s^−1^. Category IV included two varieties, FN94-0744 and GT96-44, with a mean PN value of 34.24 *μ*molm^−2^ s^−1^. The 17 sugarcane varieties could be classified into three groups based on the *E* values. Group I (high *E*) included 12 varieties, namely, MT70-611, FN94-0744, GT96-211, ROC10, YT96-107, YT96-835, FN94-0403, FN95-1726, FN96-0907, FN98-10100, YT96-794, and YT96-86, with a mean *G* value of 4.11 mmolm^−2^ s^−1^. Group II (mid-*E*) included four middle *E* varieties (GT94-116, GT95-118, GT97-18, and YT92-1287) with a mean *G* value of 3.65 mmolm^−2^ s^−1^. The variety GT96-44 alone represented Group III (low *E*) with a *G* value of only 3.10 mmolm^−2^ s^−1^. The 17 sugarcane varieties were classifiable into four categories based on GS. Category I (high GS) included five varieties (GT96-211, ROC10, YT96-107, YT96-794, and YT96-86) with a mean GS value of 154.85 mmolm^−2^ s^−1^. Category II (mid-GS) included six varieties (FN94-0403, FN95-1726, FN96-0907, FN98-10100, MT70-611, and YT96-835) with a mean GS value of 142.4 mmolm^−2^ s^−1^. Category III (low GS) included five varieties (FN94-0744, GT94-116, GT95-118, GT97-18, and YT92-1287), with a mean GS value of 128.46 mmolm^−2^ s^−1^. The variety GT96-44 alone formed Category IV with the lowest GS value of 109.7 mmolm^−2^ s^−1^.

**Figure 1:**
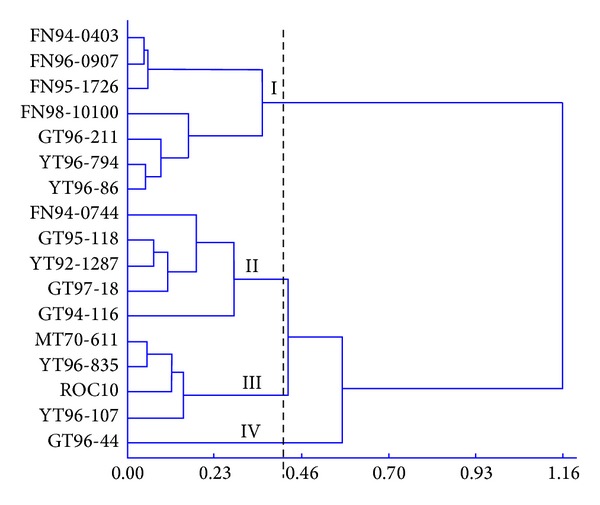
A clustering dendrogram based on photosynthetic parameters of 17 sugarcane varieties.

The result of cluster analysis showed that these 17 varieties could be grouped into four clusters (Table 3, Figure 1). Cluster I included seven varieties, namely, FN94-0403, FN95-1726, FN96-0907, FN98-10100, GT96-211, YT96-794, and YT96-86, which shared relatively high PN, *E*, and GS values. Cluster II included five varieties, that is, FN94-0744, GT94-116, GT95-118, GT97-18, and YT92-1287. The PN values of these varieties were at middle levels, while all had high *E* and GS values. Cluster III included four varieties (MT70-611, ROC10, YT96-107, and YT96-835) with middle PN, low *E*, and low GS values. Cluster IV only had one variety, GT96-44, with low values of PN, *E*, and GS.

### 3.3. Differences in the Canopy Parameters among 17 Sugarcane Varieties

There were highly significant differences in LAI, MFIA, TD, TR, and LD among the 17 sugarcane varieties at the Early Elongation stage (Table 4). According to LAI, 17 sugarcane varieties were classified into three categories. Category I included six large LAI varieties FN94-0403, GT94-116, FN96-0907, FN98-10100, GT96-44, and MT70-611, with a mean value 1.35. Category II included 4 middle LAI varieties FN94-0744, GT96-211, GT97-18, and YT92-1287, with a mean value 1.155. Category III included seven low LAI varieties FN95-1726, GT95-118, ROC10, YT96-107, YT96-794, YT96-835, and YT96-86, with a mean value 1.033.

**Figure 2:**
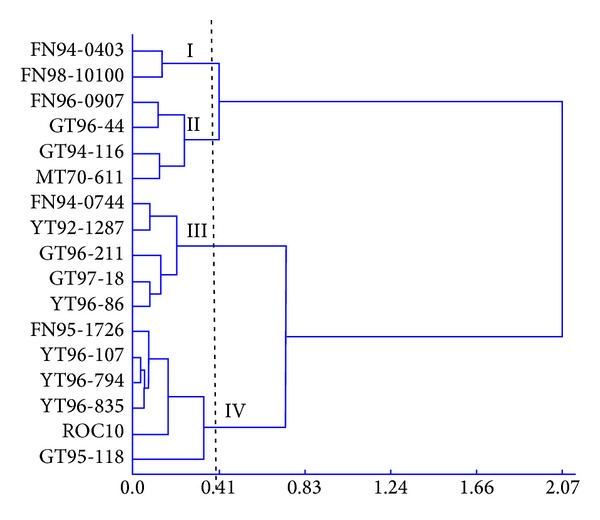
A clustering dendrogram based on canopy parameters of 17 sugarcane varieties.

Hierarchical cluster analysis was performed by merging all canopy parameters' data, that is, LAI, MFIA, TD, TR, and LD, and the output was shown in Figure 2. The 17 varieties again were grouped into four clusters (Table 5, Figure 2). Cluster I included two varieties, FN94-0403 and FN98-10100, with large mean LAI and relatively small mean MFIA and TD values. Cluster II included four varieties, namely, FN96-0907, GT94-116, GT96-44, and MT70-611. Cluster III contained five varieties, that is, FN94-0744, GT96-211, GT97-18, YT92-1287, and YT96-86, with medium LAI, MFIA, TD, TR, and LD values. Cluster IV had six varieties, namely, FN95-1726, ROC10, YT96-107, YT96-835, GT95-118, and YT96-794, with small LAI values but relatively large MFIA and TD values.

### 3.4. Relationships between Photosynthetic and Canopy Parameters and Cane Yield

Cane yield-related trait values including photosynthetic parameters (PN, *E*, and GS), canopy parameters (LAI, MFIA, TD, TR, and LD), and other traits (NSH, SSW) corresponding to the first three principal factors, joint degree (JD), and special variances (SV) are listed in Table 6. The cumulative contribution rates of the characteristic values of the first three principal factors reached 85.77%. Table 6 also indicates that the essential information of the first three principal factors can reflect most of the information of these photosynthetic gas exchange parameters, canopy parameters, and yield-related traits. The nine traits had much in common with a high degree of freedom. It also revealed that these three principal factors represented the nine traits well.

The factor loading matrices after varimax rotation in three principal factors were shown in Table 7. By comparison between the factor loading matrices of postvarimax rotation and the ones of prevarimax rotation, the load values of important variables in the principal factors were increased significantly. The biological significance of the principal factors after the varimax rotation also became more significant. As can be seen from Table 7, the large load values for canopy parameters, LAI, MFIA, TD, TR, and LD, indicated that these values played a major role in the first principal factor. The canopy parameters also had a close relationship with cane yield. On the other hand, the load values of the photosynthetic parameters PN, *E*, and GS were relatively large in the second principal factor, while the load values of SSW and NSH were relatively large in the third principal factor. Collectively, these factors were entitled as the yield component factors.

The correlation analyses data listed in Table 8 indicated the following: (1) PN had a positive correlation with *E* and GS; (2) LAI had a negative correlation with MFIA, TD, and TR, but a significantly positive correlation with NSH; (3) cane yield had a significantly negative correlation with MFIA, TD, and TR, but a significantly positive correlation with LD; and (4) SSW had significantly negative correlation with NSH, but a significantly positive correlation with  *D*. Based on regression analysis, cane yield was predictable based on photosynthetic and canopy parameters. The regression equation is as follows: Cane yield = − 27.19 − 1.69 × PN + 0.17 ×  *E* + 90.43 × LAI − 408.81 × LD + 0.0015 × NSH + 101.38 ×  *D* (*R* ^2^ = 0.928**). The relative error of cane yield fitting was less than 5% for all the 17 sugarcane varieties. Parameters NSH and *D* play a decisive role in cane yield formation with highly significant correlation coefficients of 0.902 and 0.94. Parameters LAI and LD also play a major role with significant partial correlation coefficients of 0.682 and 0.624. However, parameters PN and *E* only influence the formation of cane yield slightly with nonsignificant partial correlation coefficients of −0.331 and 0.298. Based on these observations, we conclude that the Early Elongation (growth) stage is critical to the life cycle of sugarcane. It is during this elongation stage that NSH and *D* start to form a canopy structure, which in turn determines the yield level of photosynthetic products. As was discussed in our previous report [21], cane yield formation could be influenced by many factors, which need to be adjusted according to canopy structure to ensure final cane yield formation.

## 4. Discussion and Conclusions

Regarding the genotypic differences in photosynthetic capacities among sugarcane varieties, the PN and GS could be affected greatly by the specific combining ability among sugarcane varieties [21]. However, the correlation coefficient between F_1_ hybrids and their parents reached a significant level in terms of general combining ability. Therefore, parental varieties with high photosynthetic efficiency should be selected in sugarcane breeding [22]. Sugarcane breeding practice demonstrates that PN and GS (water use efficiency) can be combined. Although the photosynthetic parameters PN, *E*, and GS had high degrees of separations in F_1_ seedling populations [17], the high photosynthetic efficiency, once generated upon cross-recombination, can be immobilized by asexual reproduction, which is a unique feature of sugarcane production. This is why all the advanced varieties possess good PN, *E*, and GS parameters [31].

In the present study, there were significant differences in the photosynthetic parameters among the 17 sugarcane varieties at the Early Elongation stage, including PN, *E*, and GS, which are in accordance with the previous reports [17, 22, 31]. The sugarcane varieties were classified according to the three photosynthetic traits, respectively, and the comprehensive traits and natural types were well reflected. The clustering patterns were relatively stable, although the masking of some traits by others and some ambiguous intercategory differences did exist. Therefore, single and integrated traits should be combined in photosynthetic efficiency breeding to obtain a better product [8].

Leaf area determines the canopy light interception. The distribution of leaf area within canopy is an important index of crop canopy structure [32]. Previous studies showed that there were significant differences in canopy parameters LAI, MFIA, and TD at the seedling stage among different genotypes. Varieties with large LAI and small TD at the seedling stage had a higher number of leaves per unit space. A positive relationship exists between even leaf spatial distribution and light interception efficiency of the population and hence higher cane yield [33]. Leaf canopy structure directly affects the canopy light interception and light distribution at different layers. Researches on morphology and anatomy revealed that sugarcane populations with transparent canopy of short, narrow, thick, and straight leaves had a larger yield potential. In addition, LAI has a very close relationship with dry matter accumulation. Therefore, a proper increase of the population LAI can increase dry matter accumulation [32–36]. In the present study, MFIA affects the receiving of solar radiation and light distribution in canopy. The ideal leaf population structure has the most effective leaf area with the characteristics of continuously changing the inclination angle distribution.

Canopy structure and light distribution of sugarcane at the Early Elongation stage play a crucial role in the accumulation and distribution of photosynthetic products, in the growth and development of population, and in the formation of final products [27–30]. This has been clearly explained by the correlations between the main canopy parameters and cane yield in this study. Zhang et al. [35] showed that canopy MFIA of winter wheat decreased with the increase of nitrogen content of soil. The light distributions of two crop populations with the same LAI are different, if the spatial distributions of MFIA and leaves are different. They can lead to different photosynthetic rates of canopy and different amounts of dry matters were produced by populations per unit time [35]. However, further optimization is still required when the yield-related traits are indirectly selected based on the main canopy parameters since the yield of sugarcane has a negative correlation with quality-related traits in this study. What should also be stressed is that, for those varieties with large LAI at the Early Elongation stage, the LAI should be adjusted to be within a certain range at the middle to late stages. Mutual shading among leaves should be avoided, which weakens light intensity, reduces light interception, and decreases photosynthetic efficiency. Therefore, rapid and accurate diagnosis of canopy structure of sugarcane and a timely, reasonable regulation of population size play important roles in the cultivation of high-quality and high-yield sugarcane varieties [32, 33].

During the Early Elongation stage, the photosynthetic and canopy parameters play a crucial role in cane yield formation [21, 25]. In this study, highly significant differences in LAI, MFIA, TD, TR, LD, PN, *E*, and GS were observed among 17 sugarcane varieties at the Early Elongation stage. However, during the factor analysis process, nine parameters were expressed by three principal factors, of which the cumulative variance contribution rate reached 85.77%. Both correlation analysis and factor analysis showed that, at the Early Elongation stage, there were close relationships between photosynthetic and canopy parameters and cane yield. Based on regression analysis for sugarcane yield, with relative error of yield fitting less than 5%, a cane yield prediction formula was established: Cane yield = − 27.19 − 1.69 × PN + 0.17 ×  *E* + 90.43 × LAI − 408.81 × LD + 0.0015 × NSH + 101.38 ×  *D* (*R* ^2^ = 0.928**), in which photosynthetic parameters, such as PN and *E*, and canopy parameters, such as LAI and LD, are used as the indirect indices for selecting better yield-related traits at the Early Elongation stage. From all the above, this study should provide the theoretical foundation and technical guidance for identifying new sugarcane varieties with high photosynthetic rates and ideal canopy structures.

## Figures and Tables

**Table 1:** Cane yield traits of 17 sugarcane varieties.

Variety	NSH	*H* */*cm	*D* */*cm	SSW*/*kg	CY*/*t ha^−1^
FN94-0403	81755 ± 2034^cde^	301.98 ± 8.91^abc^	2.85 ± 0.04^bcde^	1.93 ± 0.02^abc^	157.33 ± 5.52^ab^
FN94-0744	68151 ± 13935^de^	293.37 ± 16.2^abcd^	3.01 ± 0.04^ab^	2.08 ± 0.05^a^	142.13 ± 31.19^abc^
FN95-1726	68690 ± 5299^de^	284.33 ± 4.98^bcd^	2.95 ± 0.06^ab^	1.94 ± 0.09^abc^	133.00 ± 4.32^abcd^
FN96-0907	74886 ± 4505^cde^	290.13 ± 10.47^abcd^	2.89 ± 0.14^bc^	1.90 ± 0.21^abc^	142.73 ± 15.49^abc^
FN98-10100	107480 ± 13756^a^	272.62 ± 12.62^d^	2.56 ± 0.02^g^	1.40 ± 0.06^ef^	150.33 ± 15.09^abc^
GT94-116	104113 ± 18667^ab^	285.3 ± 10.9^bcd^	2.30 ± 0.13^h^	1.19 ± 0.18^f^	121.77 ± 5.96^bcd^
GT95-118	65862 ± 3591^e^	310.63 ± 21.9^ab^	2.70 ± 0.06^efg^	1.78 ± 0.09^bcd^	117.23 ± 11.46^cd^
GT96-211	72192 ± 15672^cde^	302.33 ± 19.74^abc^	2.75 ± 0.05^cdef^	1.79 ± 0.11^bcd^	130.37 ± 34.78^abcd^
GT96-44	86738 ± 8323^bcd^	282.75 ± 13.89^cd^	2.64 ± 0.09^fg^	1.55 ± 0.13^de^	134.20 ± 8.94^abcd^
GT97-18	81890 ± 3484^cde^	314.48 ± 18.44^a^	2.64 ± 0.04^fg^	1.72 ± 0.06^cd^	140.73 ± 9.85^abc^
MT70-611	70441 ± 8782^cde^	303.88 ± 8.48^abc^	2.73 ± 0.05^*def*^	1.78 ± 0.11^bcd^	124.57 ± 8.21^bcd^
ROC10	70037 ± 9851^cde^	280.83 ± 9.21^cd^	2.55 ± 0.12^g^	1.44 ± 0.16^e^	100.40 ± 14.09^d^
YT92-1287	74347 ± 7740^cde^	287.03 ± 6.94^bcd^	2.87 ± 0.11^bcd^	1.86 ± 0.18^abc^	137.40 ± 8.14^abc^
YT96-107	80812 ± 16699^cde^	287.58 ± 24.33^bcd^	2.54 ± 0.09^g^	1.47 ± 0.2^e^	120.57 ± 37.88^bcd^
YT96-794	62764 ± 5456^e^	279.97 ± 13.09^cd^	2.93 ± 0.05^ab^	1.89 ± 0.13^abc^	118.63 ± 13.96^cd^
YT96-835	88759 ± 3266^bc^	270.3 ± 9.88^d^	2.72 ± 0.11^*def*^	1.57 ± 0.16^de^	139.63 ± 12.64^abc^
YT96-86	81216 ± 9134^cde^	271.8 ± 5.42^d^	3.06 ± 0.14^a^	2.00 ± 0.19^ab^	163.53 ± 31.03^a^

Notes: (1) NSH: stalk number per hectare; *H*: plant height; *D*: stalk diameter; SSW: fresh single stalk weight; CY: cane yield. (2) Different letters in the same column indicate significant difference among different treatments at 0.05 level (Duncan Test).

**Table 2:** Photosynthesis rate (PN), transpiration rate (*E*), and stomatal conductivity (GS) of 17 sugarcane varieties.

Variety	PN*/μ*molm^−2^ s^−1^ CO_2_	E*/*mmolm^−2^ s^−1^ H_2_O	GS*/*mmolm^−2^ s^−1^
FN94-0403	36.09 ± 5.75^abcd^	4.38 ± 0.83^a^	143.97 ± 38.35^bcde^
FN94-0744	33.23 ± 7.33^de^	3.98 ± 1.06^abcde^	128.90 ± 45.75^*def*^
FN95-1726	35.5 ± 6.25^abcde^	4.35 ± 0.85^ab^	139.54 ± 41.69^bcdef^
FN96-0907	35.57 ± 5.70^abcde^	4.36 ± 0.78^ab^	146.03 ± 49.61^abc^
FN98-10100	37.53 ± 8.40^a^	4.24 ± 1.05^abc^	140.55 ± 47.46^bcdef^
GT94-116	36.66 ± 6.89^abc^	3.68 ± 1.30^ef^	126.61 ± 50.49^f^
GT95-118	33.87 ± 7.38^cde^	3.62 ± 0.97^ef^	125.37 ± 45.27^f^
GT96-211	37.34 ± 7.36^ab^	3.96 ± 1.09^bcde^	161.28 ± 67.16^a^
GT96-44	32.56 ± 5.77^e^	3.10 ± 1.10^g^	109.70 ± 43.36^g^
GT97-18	34.47 ± 5.57^abcde^	3.76 ± 0.74^*def*^	134.12 ± 30.17^cdef^
MT70-611	34.39 ± 7.78^abcde^	3.93 ± 1.03^cdef^	144.71 ± 59.02^abcd^
ROC10	34.00 ± 6.19^bcde^	3.94 ± 0.60^cde^	155.39 ± 42.52^ab^
YT92-1287	34.59 ± 8.32^abcde^	3.54 ± 1.35^f^	127.32 ± 51.43^ef^
YT96-107	35.56 ± 7.09^abcde^	3.93 ± 1.14^cdef^	150.28 ± 49.79^abc^
YT96-794	37.04 ± 7.36^abc^	4.10 ± 0.88^abcd^	156.77 ± 53.82^ab^
YT96-835	34.14 ± 7.26^abcde^	4.02 ± 0.74^abcde^	139.60 ± 43.54^bcdef^
YT96-86	36.90 ± 6.78^abc^	4.14 ± 0.62^abcd^	150.54 ± 41.82^abc^

Notes: (1) PN: photosynthesis rate, *μ*molm^−2^ s^−1^; *E*: transpiration rate, mmolm^−2^ s^−1^; GS: stomatal conductance, mmolm^−2^ s^−1^. (2) Different letters in the same column indicate significant difference among different treatments at 0.05 level (Duncan test).

**Table 3:** Differences among different photosynthetic gas exchange parameters among 17 sugarcane varieties.

Cluster	PN	*E *	GS	Variety
I	36.58	4.22	148.38	FN94-0403, FN95-1726, FN96-0907, FN98-10100, GT96-211, YT96-794, YT96-86
II	34.52	3.96	147.50	FN94-0744, GT94-116, GT95-118, GT97-18, YT92-1287
III	34.56	3.72	128.46	MT70-611, ROC10, YT96-107, YT96-835
IV	32.56	3.1	109.7	GT96-44

Notes: PN: photosynthesis rate, *μ*molm^−2^ s^−1^; *E*: transpiration rate, mmolm^−2^ s^−1^; GS: stomatal conductance, mmolm^−2^ s^−1^.

**Table 4:** Difference of canopy parameters among 17 sugarcane varieties.

Variety	LAI	MFIA	TD	TR	LD
FN94-0403	1.487 ± 0.230^a^	43.27 ± 13.59^c^	0.33 ± 0.06^ab^	0.277 ± 0.16^f^	0.783 ± 0.08^ab^
FN94-0744	1.160 ± 0.214^bcde^	50.56 ± 11.90^bc^	0.39 ± 0.07^ab^	0.354 ± 0.19^cde^	0.748 ± 0.09^abc^
FN95-1726	1.050 ± 0.072^de^	62.94 ± 6.55^ab^	0.44 ± 0.02^ab^	0.410 ± 0.23^abc^	0.722 ± 0.09^bc^
FN96-0907	1.247 ± 0.064^abcde^	55.99 ± 12.51^abc^	0.37 ± 0.02^ab^	0.351 ± 0.20^cde^	0.770 ± 0.09^ab^
FN98-10100	1.420 ± 0.035^ab^	50.19 ± 9.93^bc^	0.32 ± 0.04^b^	0.291 ± 0.16^ef^	0.799 ± 0.06^a^
GT94-116	1.323 ± 0.086^abcd^	49.82 ± 4.23^bc^	0.37 ± 0.04^ab^	0.325 ± 0.17^*def*^	0.761 ± 0.11^ab^
GT95-118	0.987 ± 0.316^e^	71.29 ± 11.89^a^	0.51 ± 0.13^a^	0.460 ± 0.28^a^	0.684 ± 0.13^c^
GT96-211	1.180 ± 0.125^bcde^	60.37 ± 4.78^abc^	0.41 ± 0.07^ab^	0.374 ± 0.21^bcd^	0.736 ± 0.07^abc^
GT96-44	1.367 ± 0.076^abc^	57.23 ± 4.90^abc^	0.37 ± 0.04^ab^	0.322 ± 0.19^*def*^	0.776 ± 0.05^ab^
GT97-18	1.127 ± 0.211^cde^	58.64 ± 13.19^abc^	0.44 ± 0.11^ab^	0.381 ± 0.23^bcd^	0.719 ± 0.10^bc^
MT70-611	1.267 ± 0.096^abcde^	43.16 ± 5.30^c^	0.37 ± 0.02^ab^	0.316 ± 0.15^*def*^	0.756 ± 0.12^abc^
ROC10	1.063 ± 0.087^de^	67.57 ± 1.03^ab^	0.47 ± 0.03^ab^	0.425 ± 0.25^ab^	0.708 ± 0.06^bc^
YT92-1287	1.153 ± 0.154^bcde^	52.96 ± 11.11^abc^	0.41 ± 0.05^ab^	0.371 ± 0.20^bcd^	0.735 ± 0.12^abc^
YT96-107	1.027 ± 0.085^e^	61.00 ± 16.68^abc^	0.45 ± 0.10^ab^	0.415 ± 0.23^abc^	0.712 ± 0.10^bc^
YT96-794	1.023 ± 0.106^e^	60.15 ± 12.02^abc^	0.44 ± 0.06^ab^	0.409 ± 0.23^abc^	0.718 ± 0.10^bc^
YT96-835	0.993 ± 0.090^e^	58.57 ± 7.32^abc^	0.45 ± 0.06^ab^	0.419 ± 0.22^abc^	0.715 ± 0.10^bc^
YT96-86	1.087 ± 0.080^de^	54.40 ± 5.16^abc^	0.43 ± 0.04^ab^	0.383 ± 0.20^bcd^	0.726 ± 0.11^abc^

Notes: (1) LAI: leaf area index; MFIA: mean leaf angle degree; TD: transmission coefficient for diffuse penetration; TR: transmission coefficient for solar beam radiation penetration; LD: leaf distribution; (2) Different letters in the same column indicate significant difference among different treatments at 0.05 level (Duncan test).

**Table 5:** Difference in different canopy parameters among 17 sugarcane varieties.

Cluster	LAI	MFIA	TD	TR	LD	Variety
I	1.454	46.730	0.325	0.284	0.791	FN94-0403, FN98-10100
II	1.301	51.550	0.370	0.329	0.766	FN96-0907, GT94-116, GT96-44, MT70-611
III	1.141	55.386	0.416	0.373	0.733	FN94-0744, GT96-211, GT97-18, YT92-1287, YT96-86
IV	1.024	63.587	0.460	0.423	0.710	FN95-1726, GT95-118, ROC10, YT96-107, YT96-794, YT96-835

Notes: LAI: leaf area index; MFIA: mean leaf angle degree; TD: transmission coefficient for diffuse penetration; TR: transmission coefficient for solar beam radiation penetration; LD: leaf distribution.

**Table 6:** Loading matrix of characters initial factor in sugarcane varieties.

Parameter	Factor 1	Factor 2	Factor 3	JD	SV
PN	0.302	0.650	0.553	0.820	0.180
*E *	0.163	0.883	0.104	0.817	0.183
GS	−0.187	0.821	0.377	0.852	0.148
LAI	0.937	−0.146	−0.011	0.899	0.101
MFIA	−0.863	−0.106	0.199	0.795	0.205
TD	−0.979	−0.028	0.040	0.960	0.040
TR	−0.977	0.050	0.079	0.963	0.037
LD	0.963	−0.046	−0.027	0.931	0.069
NSH	0.645	−0.325	0.534	0.806	0.194
SSW	−0.117	0.557	−0.807	0.976	0.024
CY	0.608	0.327	−0.374	0.616	0.384
VC	5.414	2.438	1.583		
%	49.220	22.160	14.390		
% CC	49.220	71.380	85.770		

Notes: PN: photosynthesis rate, *μ*molm^−2^ s^−1^; *E*: transpiration rate, mmolm^−2^ s^−1^; GS: stomatal conductance, mmolm^−2^ s^−1^; LAI: leaf area index; MFIA: mean leaf angle degree; TD: transmission coefficient for diffuse penetration; TR: transmission coefficient for solar beam radiation penetration; LD: leaf distribution; CY: cane yield; NSH: number of stalks per hectare; SSW: single stalk weight; VC: variance contribution; CC: cumulative contribution; JD: joint degree; SV: special variance; ^−^negative load value; the larger absolute value means the larger load value.

**Table 7:** Loading matrix of varimax orthogonal rotation factor in sugarcane varieties.

Parameter	Factor 1	Factor 2	Factor 3	JD	SV
PN	0.204	0.854	−0.221	0.820	0.180
*E *	0.155	0.838	0.301	0.817	0.183
GS	−0.241	0.886	0.097	0.852	0.148
LAI	0.920	−0.064	−0.222	0.899	0.101
MFIA	−0.886	−0.064	−0.073	0.795	0.205
TD	−0.969	−0.079	0.122	0.960	0.040
TR	−0.974	0.008	0.124	0.963	0.037
LD	0.950	0.019	−0.165	0.931	0.069
NSH	0.528	0.013	−0.726	0.806	0.194
SSW	0.046	0.103	0.981	0.976	0.024
CY	0.673	0.158	0.372	0.616	0.384
VC	5.278	2.267	1.890		
% CC	47.982	68.588	85.768		

Notes: PN: photosynthesis rate, *μ*molm^−2^ s^−1^; *E*: transpiration rate, mmolm^−2^ s^−1^; GS: stomatal conductance, mmolm^−2^ s^−1^; LAI: leaf area index; MFIA: mean leaf angle degree; TD: transmission coefficient for diffuse penetration; TR: transmission coefficient for solar beam radiation penetration; LD: leaf distribution; CY: cane yield; NSH: number of stalks per hectare; SSW: single stalk weight; VC: variance contribution; CC: cumulative contribution; JD: joint degree; SV: special variance; ^−^negative load value; the larger absolute value means the larger load value.

**Table 8:** Correlation matrix among the traits in sugarcane varieties.

Parameter	PN	*E *	GS	LAI	MFIA	TD	TR	LD	NSH	*D *	SSW	CY
PN	1.000											
*E *	0.565∗	1.000										
GS	0.608∗∗	0.679∗∗	1.000									
LAI	0.176	0.014	−0.258	1.000								
MFIA	−0.192	−0.187	0.099	−0.739∗∗	1.000							
TD	−0.273	−0.181	0.142	−0.933∗∗	0.851∗∗	1.000						
TR	−0.206	−0.085	0.201	−0.963∗∗	0.878∗∗	0.978∗∗	1.000					
LD	0.220	0.133	−0.204	0.942∗∗	−0.769∗∗	−0.986∗∗	−0.957∗∗	1.000				
NSH	0.290	−0.083	−0.284	0.549∗	−0.390	−0.533∗	−0.529∗	0.561∗	1.000			
*D *	0.006	0.404	0.195	−0.219	−0.078	0.057	0.105	−0.089	−0.600	1.000		
SSW	−0.056	0.349	0.151	−0.176	−0.093	0.069	0.081	−0.116	−0.689∗∗	0.953∗∗	1.000	
CY	0.248	0.340	−0.100	0.422	−0.551∗	−0.522∗	−0.510∗∗	0.503∗	0.342	0.484	0.428	1.000

Notes: (1) PN: photosynthesis rate, *μ*molm^−2^ s^−1^; *E*: transpiration rate, mmolm^−2^ s^−1^; GS: stomatal conductance, mmolm^−2^ s^−1^; LAI: leaf area index; MFIA: mean leaf angle degree; TD: transmission coefficient for diffuse penetration; TR: transmission coefficient for solar beam radiation penetration; LD: leaf distribution; CY: cane yield; NSH: number of stalks per hectare; *D*: diameter; SSW: fresh single stalk weight. (2) ^−^Negative load value; the larger absolute value means the larger load value. (3) ∗Significant differences at the level of 0.05; ∗∗significant differences at the level of 0.01.

## References

[1] Wang KR, Li SK, Song GJ, Chen G, Cao SZ (2002). Studies on cultivated physiological indexes for high-yielding cotton in Xinjiang. *Scientia Agricultura Sinica*.

[2] Zhao M, Li JG, Zhang B, Dong ZQ, Wang MY (2006). The compensatory mechanism in exploring crop production potential. *Acta Agronomica Sinica*.

[3] Peng SB, Khush GS, Virk P, Tang Q, Zou Y (2008). Progress in ideotype breeding to increase rice yield potential. *Field Crops Research*.

[4] Hua S, Yuan S, Shamsi IH (2009). A comparison of three isolines of cotton diff ering in fiber color for yield, quality, and photosynthesis. *Crop Science*.

[5] Feng GY, Luo HH, Yao YD (2012). Spatial distribution of leaf and boll in relation to canopy photosynthesis of super high-yielding cotton in xinjiang. *Scientia Agricultura Sinica*.

[6] Long SP, Zhu X-G, Naidu SL, Ort DR (2006). Can improvement in photosynthesis increase crop yields?. *Plant, Cell and Environment*.

[7] Jin LB, Zhang JW, Li B (2013). Canopy structure and photosynthetic characteristics of high yield and high nitrogen efficiency summer maize. *Scientia Agricultura Sinica*.

[8] Zhao M, Wang SA, Wang MY, Li SK (1999). Cluster analysis for photosynthetic characters of inbred lines of maize in china. *Acta Agronomica Sinica*.

[9] Guo XQ, Zhao M, Li SK (1997). Studies on photosynthetic properties of different maize inbred lines. *Maize Science*.

[10] Li X, Yan JM, Ji BH, Jiao DM (1999). Varietal difference in photosynthetic characteristics of rice under photooxidation and shading. *Acta Agronomica Sinica*.

[11] Sinclair TR, Sheehy JE (1999). Erect leaves and photosynthesis in rice. *Science*.

[12] Zhai HQ, Cao SQ, Tang YL (2002). Analysis on combining ability and heritability of photosynthetic characters in indica hybrid rice. *Acta Agronomica Sinica*.

[13] Du WG, Zhang GR, Man WQ (2001). Development of soybean cultivars (germplasm) with high photosynthetic efficiency (HPE) and rediscussion of breeding for HPE. *Soybean Science*.

[14] Li WH, Lu QT, Hao NB, Zhang QD, Ge QY, Kuang TY (2000). The high photosynthetic efficiency characteristics of high-yield varieties in soybean. *Acta Biophysica Sinica*.

[15] Zhang MQ, Chen RK, Luo J, Lu JL, Xu JS (2000). Analyses for inheritance and combining ability of photochemical activities measured by chlorophyll fluorescence in the segregating generation of sugarcane. *Field Crops Research*.

[16] Gao SJ, Chen RK, Zhang MQ, Liao JF (1999). Genetic variation of net photosynthetic rate in sugarcane hybrid progenies. *Journal of Fujian Agricultural University*.

[17] Lü JL, Chen RK, Zhang MQ, Luo J, Lin YQ, Fang WC (2000). Study on the genetic analysis of parents with high photosynthetic efficiency in sugarcane. *Scientia Agricultura Sinica*.

[18] Zhang MQ, Lü JL, Chen RK (1998). Diurnal variation of photosynthetic rate in sugarcane and its responses to light and temperature. *Journal of Fujian Agricultural University*.

[19] Xing YX, Yang LT, Li YR (2002). Effect of ethephon on respiratory exchange in different sugarcane varieties. *Chinese Journal of Tropical Crops*.

[20] Lü JL, Chen RK, Zhang MQ, Li CM, Liao JF (1998). Seasonal change of the net photosynthesis rate, chlorophyll content and specific weight of leaf of sugarcane and their relationships. *Journal of Fujian Agricultural University*.

[21] Luo J, Que YX, Zhang H, Xu LP (2013). Seasonal variation of the canopy structure parameters and its correlation with yield-related traits in sugarcane. *The Scientific World Journal*.

[22] Luo J, Zhou H, Zhang MQ, Chen RK, Zhang H (2004). Genetic analysis of main economic and photosynthetic traits in energy sugarcane. *Chinese Journal of Applied & Environmental Biology*.

[23] Zhao HJ, Zou Q, Guo TC, Yu ZW, Wang YH (2002). Regulating effects of density and top-dressing time of nitrogen on characterstics of radiation transmission and photosynthesis in canopy of massive-spike winter wheat variety L906. *Acta Agronimica Sinica*.

[24] Gao SJ, Luo J, Chen RK, Lü JL (2002). Mathematical analysis on leaf morphology of sugarcane varieties. *Journal of Plant Genetic Resources*.

[25] Chen RK, Xu LP, Lin YQ (2011). *Modern Sugarcane Genetic Breeding*.

[26] Tan ZW, Liang JN, Chen JP, Chen PS (2001). Studies on the relationship of morphological, anatomical characters in seedling stage and juice sugar, yield on sugarcane genotypes. *Journal of South China Agricultural University*.

[27] Zhang MQ, Chen RK, Gao SJ, Lü JL, Fang F, Xu NN (1997). Morphophysiological responses of sugarcane genotypes to water stress. *Scientia Agricultura Sinica*.

[28] Gao SJ, Chen RK, Zhang H, Xu NN, Deng ZH, Luo J (2006). Factor and clustering analysis of economic characters in sugarcane(Saccharum spp.). *Journal of Fujian Agriculture and Forestry University*.

[29] Zhang RH, Chen TH, Zheng YF, Wang QM (1998). Interspecific variation in physiology of conifer and analysis by fuzzy cluster. *Journal of Nanjiang Forestry University*.

[30] Tang QY, Zhang CX (2013). Data Processing System (DPS) software with experimental design, statistical analysis and data mining developed for use in entomological research. *Insect Science*.

[31] Luo J, Wang Q, Zhang H, Lin Y, Chen Y (2007). Phenetic classification for photosynthetic characters of different sugarcane varieties. *Chinese Journal of Applied and Environmental Biology*.

[32] Luo J, Zhang H, Deng Z (2005). Relationship between canopy characters and leaf morphology at different positions of sugarcane. *Chinese Journal of Applied and Environmental Biology*.

[33] Luo J, Zhang H, Lin YQ, Deng ZH, Chen RK (2004). The Relationship of canopy structure characters in the seedling and yield characters on sugarcane genotypes. *Chinese Journal of Tropical Crops*.

[34] Zhang YQ, Yang HS, Gao JL (2011). Study on canopy structure and physiological characteristics of super-high yield spring maize. *Scientia Agricultura Sinica*.

[35] Zhang XY, Du JD, Zheng DF (2011). Effect of density on canopy structure and photosynthetic characteristics in soybean population. *Agricultural Research in the Arid Areas*.

[36] Zhang YM, Li JS, Qian WP (1996). Canopy structure and light distribution in winter wheat. *Acta Agriculturae Boreali-Sinica*.

